# Gene-nutrient interactions and susceptibility to human obesity

**DOI:** 10.1186/s12263-017-0581-3

**Published:** 2017-10-30

**Authors:** Joseph J. Castillo, Robert A. Orlando, William S. Garver

**Affiliations:** Department of Biochemistry and Molecular Biology, School of Medicine, University of New Mexico Health Sciences Center, Albuquerque, NM 87131-0001 USA

**Keywords:** Energy balance, Gene-nutrient, Heritability, Metabolism, Obesity

## Abstract

A large number of genome-wide association studies, transferability studies, and candidate gene studies performed in diverse populations around the world have identified gene variants that are associated with common human obesity. The mounting evidence suggests that these obesity gene variants interact with multiple environmental factors and increase susceptibility to this complex metabolic disease. The objective of this review article is to provide concise and updated information on energy balance, heritability of body weight, origins of gene variants, and gene-nutrient interactions in relation to human obesity. It is proposed that knowledge of these related topics will provide valuable insight for future preventative lifestyle intervention using targeted nutritional and medicinal therapies.

## Background

**Fig. 1:**
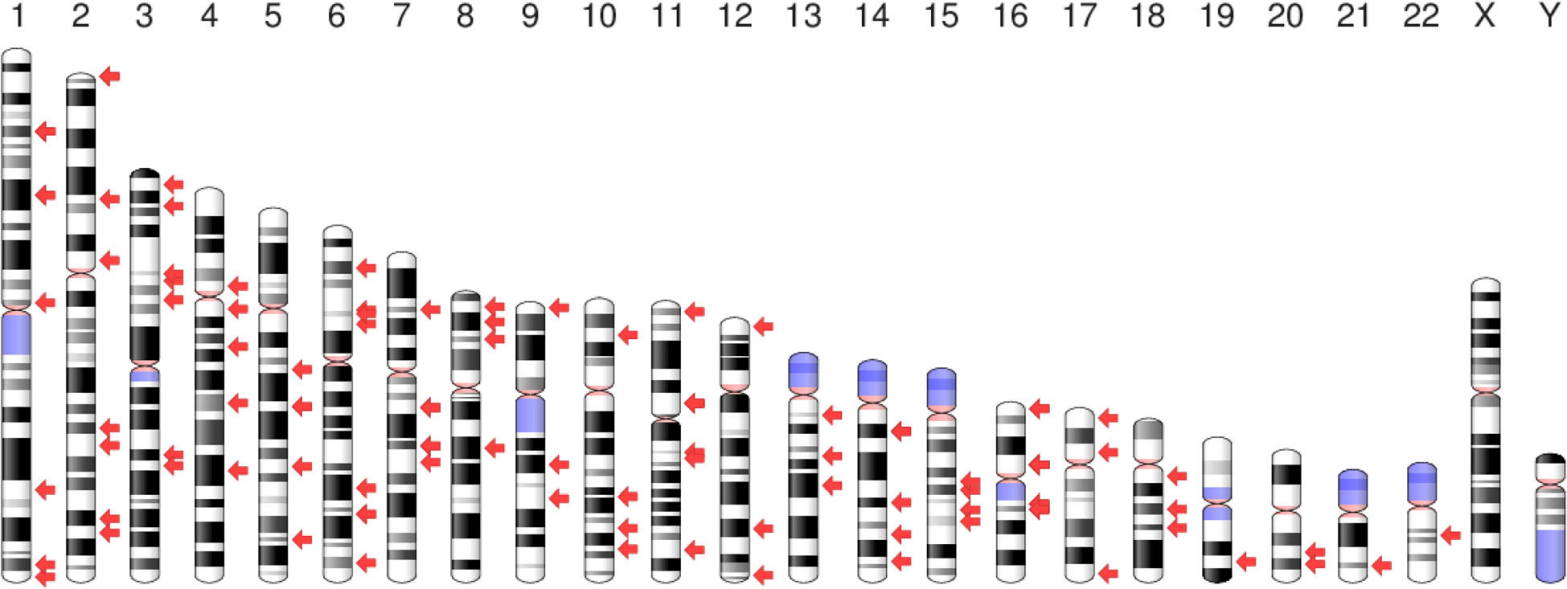
Chromosomal ideogram of human obesity susceptibility genes. The chromosomal loci for 127 obesity susceptibility genes are provided using a chromosomal ideogram and denoted by red arrows

The most recent report from the Centers for Disease Control and Prevention indicates that 16.9% of children and adolescents (2 to 19 years of age) and 34.9% of adults (20 years of age and older) have obesity as defined by a body mass index (BMI) greater than the age-adjusted 95th percentile and 30 kg/m^2^, respectively [1]. To determine the molecular basis for obesity that has reached epidemic proportions in the USA and other developed countries, a large number of genome-wide association studies (GWAS) have been performed to identify obesity gene variants that increase susceptibility to this common metabolic disease [2–14]. These GWAS and subsequent transferability studies, in addition to candidate gene studies performed in diverse populations around the world, have identified gene variants that are associated with common human obesity [6, 15–18]. To date, approximately 140 obesity susceptibility genes have been found to be associated with measures of adiposity (BMI, body fat percentage, and/or waist circumference), and the recent progress is being made to define the pathophysiology of human of human obesity [19]. A chromosomal ideogram showing the loci of these obesity susceptibility genes is provided (Fig. 1). The obesity epidemic is a recent manifestation that has occurred during the past few decades; and not all individuals or populations are adversely affected, thereby suggesting differences based on genetic variability and interaction with environmental factors [20–22]. The combined environmental factors comprising the “obesogenic environment” include dietary nutrients, age, gender, ethnicity, duration of sleep, amount of physical activity, sedentary behavior, stress, smoking, alcohol consumption, use of medication, and depression. It is generally accepted that, of these environmental factors, a primary cause for susceptibility to obesity is through gene-nutrient interactions. The importance of gene-nutrient interactions in promoting obesity and other complex metabolic diseases is evidenced by the rapidly emerging scientific disciplines of nutritional genetics (nutrigenetics) and nutritional genomics (nutrigenomics) that may provide more effective personalized healthcare [23]. It should be noted that a number of brief review articles that focus on gene and environment interactions in relation to obesity have recently been published [22, 24–26].

## Energy balance

The cause of weight gain has often been considered in terms of positive energy balance; the components of which include increased energy intake, decreased energy output, and energy deposition [27]. When energy in the form of calories from food and drink is greater than energy output resulting from (i) resting metabolic rate, (ii) absorption and metabolism of dietary nutrients, (iii) heat production or thermogenesis, and (iv) physical activity, a state of positive energy balance results to promote deposition of triacylglycerol within adipose tissue. In contrast, when energy in the form of calories from food and drink is less than energy output, a state of negative energy balance results to promote lipolysis of triacylglycerol and mobilization of fatty acids from adipose tissue. It should be noted that although the physical laws of thermodynamics provide exact values for these energy transformations, the relative sign and magnitude of these values does not provide information concerning the physiological basis responsible for changes in energy balance [28, 29]. Moreover, although incapable of being measured using current technology, mathematical models have predicted that even small energy surpluses or deficits (~1%) over time results in weight gain or weight loss, respectively [28, 30].

## Heritability of body weight and interaction with environmental factors

A study performed by Sir Francis Galton over 100 years ago provided the first evidence suggesting that measures of growth (height and body weight) may be heritable [31]. Since that time, the Hereditary Abilities Study, which represented the first comprehensive study performed in the USA to investigate heritability of physical traits, indicated that the greater part for variance of heritability was genetic [32]. This finding was consistent with several other studies, indicating a high heritability of body weight among twins that may or may not have been influenced by interaction with the environment [33–35]. A more recent study has reported that both BMI and waist circumference are of high heritability (77%), with only modest environmental effects for children living in an obesogenic environment [36]. With respect to the variance of heritability for BMI, a systematic review of twin studies (140,525 twins) and family studies (42,968 family members) indicated a wide range (47 to 90%) of heritability resulting from population differences [37]. The population differences affecting variance of heritability for BMI has stimulated an interest in understanding how obesity susceptibility genes interact with environmental factors to increase weight gain, in what has formally become known as “gene-environment interactions,” defined as a response or adaptation to an environmental agent, a behavior, or a change in behavior, conditional to the genotype of the individual [38]. For example, a study using 12 monozygotic twins who consumed a 1000 kcal/day surplus of calories for a period of 100 days while maintaining a sedentary lifestyle demonstrated a gene-nutrient interaction in relation to weight gain [39]. The results from this study showed a significant within twin-pair resemblance in adaptation to the excess calories (≥3 times more variance in response between twin-pairs than within twin-pairs) indicating that genetic susceptibility must have influenced the weight gain. Moreover, a study designed to assess genetic and environmental influences using 114 monozygotic twins, 81 dizygotic twins, and 98 virtual twins (same age but unrelated siblings) indicated that genetic susceptibility contributed ~65% to heritability for BMI [40]. The heritability of childhood obesity was also closely examined and confirmed in a study using 8234 children that demonstrated a fourfold increase in risk for childhood obesity if one parent was obese and a tenfold increase risk for childhood obesity if both parents were obese [41]. Therefore, it is now accepted that heritability can contribute an estimated 40–70% to the variation of BMI within populations [42]. It should also be noted that genetic architecture for different forms of obesity, whether rare Mendelian (syndromic and non-syndromic) or non-Mendelian (common) obesity, are believed to display variable phenotypes due to interactions with other genes [43–45].

## Missing heritability

The “common disease, common variant” hypothesis states that genetic risk for common diseases is due to variants of high frequency [46, 47]. It was once thought that common variants of high frequency would explain common disease heritability, defined as the proportion of phenotypic variance in a population due to additive genetic factors. However, after identifying hundreds of different variants associated with common diseases, the combination of variants were found to account for only a small proportion of the estimated heritability [48]. In other words, although the estimated heritability for BMI within populations was 40–70% (heritability inferred indirectly from population data and provided in the denominator), only a few percent of the actual heritability was accounted for through the combined phenotypic effect size or penetrance of known variants (heritability determined directly from effect sizes and provided in the numerator) [11, 49]. Thus, there was increasing concern that identification of “missing heritability,” or what has more appropriately been termed “hidden heritability,” would be necessary to understand how variants contribute to common diseases for successful translation of this information into clinical practice [50–52]. It is speculated that the identified GWAS variants may be in partial linkage-disequilibrium with low-frequency variants with larger phenotypic effect sizes, thereby accounting for the missing heritability [53–55]. Although some low-frequency variants of intermediate effect size were found to exist in partial linkage-disequilibrium with GWAS variants, other studies suggest that these rare variants are unlikely to account for the missing heritability [56–60]. Moreover, it was proposed that the amount of missing heritability was overestimated and therefor represented as “phantom heritability” and that unidentified gene interactions (gene-gene and gene-nutrient) may account for any remaining heritability [61]. Finally, in hopes of resolving the case of missing heritability for common diseases, studies now indicate that imputed variants are capable of accounting for a large proportion (~30%) of the estimated heritability for body weight, with the remaining proportion likely accounted for by unidentified gene interactions [62, 63].

## Thrifty and drifty genotype hypotheses

Consistent with obesity susceptibility gene variants interacting with an obesogenic environment to increase obesity, James V. Neel proposed the thrifty genotype hypothesis based on positive selection (adaptation) of “thrifty genes” resulting from seasonal food shortages and episodic famines during human evolution [64, 65]. A number of years later, John R. Speakman proposed the drifty genotype hypothesis based on neutral selection (genetic drift) of “drifty genes” resulting from predation release characterized by liberation from predation pressures due to the advent of fire and development of more advanced weapons technology, thereby, in effect, increasing the upper limit of body weight set points [66, 67]. In support of both these hypotheses, commentaries published by experts in the field suggest that positive and neutral selection of gene variants has occurred during human evolution to optimize efficient storage of food energy for later use when food becomes limiting [68–70]. This commentary is also evidenced by a recent study performed with 9416 individuals in 14 European countries indicating that although environmental differences masked genetic differentiation for BMI, it was determined that 8% of the captured additive genetic variance for BMI was reflected in population genetic differences [71]. However, some studies have questioned the thrifty genotype hypothesis due to the high prevalence of obesity within certain populations, such as those populations in the South Pacific, because past seasonal food shortages and episodic famines are believed not to have occurred, and that a recent comprehensive study performed using signatures of positive selection at obesity susceptibility gene variants did not find evidence for the “thrifty gene” hypothesis [72, 73]. However, other studies have provided evidence of “thrifty gene variants” present within the CREBFR gene among Samoans and the PPARGC1A gene among Tongans in the South Pacific, thereby explaining high rates of obesity among these two populations [8, 74]. Therefore, it is anticipated that the intellectual debate concerning origin of obesity susceptibility genes will continue.

## Dietary macronutrients

The obesogenic environment consists of a complex interplay of contributing factors that influence behavior effecting dietary choice, physical activity, and/or metabolism responsible for maintaining energy balance [75]. A number of studies suggest that both sedentary behavior (viewing television, playing video games, doing cognitive work, and listening to music) and reduced overall physical activity in addition to shorter sleep duration promote the overconsumption of dietary nutrients, particularly fats and refined carbohydrates [76–80]. The increased consumption of a high-fat diet, particularly a high-fat diet enriched with saturated fatty acids, has been found to be strongly associated with increased adiposity in children [81, 82]. Moreover, another recent study performed using 810 participants indicated a highly significant association of saturated fatty acid consumption (but not plant protein, carbohydrates, or other types of fat) at 6 months with body weight at 18 months of age [83]. Consistent with these results, obesity susceptibility genes have been reported to preferentially interact with saturated fatty acids, but not monounsaturated fatty acids or polyunsaturated fatty acids, to promote weight gain [84, 85]. For these reasons, it is widely accepted that high-fat diets, characterized by enhanced palatability and high energy density, may have a primary role for the obesity epidemic. However, it should also be noted that increased consumption of carbohydrates, particularly refined carbohydrates and sugar-sweetened beverages, during the past 30 years also parallels the increased prevalence of obesity [86–88].

## Susceptibility genes for human obesity

Over the course of the decade (1996–2005) preceding GWAS, an extensive amount of work was performed to identify susceptibility genes for human obesity. The culmination of these studies resulted in the identification of 140 candidate susceptibility genes [89]. However, to date only a limited number (~25%) of these obesity susceptibility genes have been validated using independent studies. These genes now represent a comprehensive list of obesity susceptibility candidates that are associated with measures of adiposity (BMI, body fat percentage, and waist circumference) [42, 90–92]. It should be noted that in most cases, the biological function of the encoded proteins and gene products derived from these obesity susceptibility genes remains undefined and therefore further studies must be performed using cells grown in culture, animal models, and diverse ethnic populations before development of targeted nutritional or medicinal therapies.

## Gene-nutrient interactions that promote obesity

**Fig. 2:**
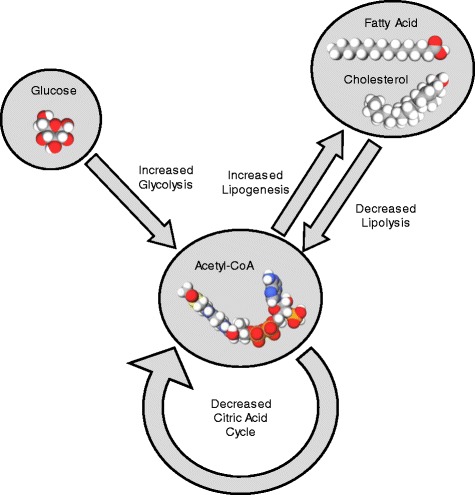
A general diagram showing the physiological basis responsible for the NPC1 gene-nutrient interaction that promotes weight gain and susceptibility to obesity. Mouse model tissues (liver and adipose) and fibroblasts grown in culture indicate (i) increased glycolysis (oxidation of glucose and conversion to pyruvate in the cytoplasm), (ii) decreased oxidative metabolism (conversion of pyruvate to acetyl-CoA and condensation with oxaloacetate to produce citrate in the mitochondria), (iii) increased lipogenesis pathway (transport of citrate from the mitochondria and conversion to acetyl-CoA and malonyl-CoA for lipid (cholesterol and fatty acid) synthesis in the cytoplasm), and (iv) decreased lipolysis pathway (transport of fatty acid from the cytoplasm and conversion of fatty acid to acetyl-CoA in the mitochondria)

The best characterized obesity susceptibility gene known to interact with a dietary nutrient that predisposes to weight gain was identified using a candidate gene approach followed by a number of population-based studies. The apolipoprotein A2 (APOA2) gene is a member of the apolipoprotein multigene family for which the encoded APOA2 protein is associated with high-density lipoprotein (HDL), which modulates activity of lipoprotein lipase to influence liver lipogenesis and adipose lipolysis [93–96]. An initial study indicated that individuals who are homozygous affected for the loss-of-function APOA2 variant (rs5082) had increased measures of adiposity (body weight, BMI, and waist circumference) characterized with increased consumption of food composed of fat and protein compared to individuals who are homozygous normal or heterozygous for this gene variant [97]. A second study identified an association between homozygous affected individuals for the same gene variant and consumption (≥22 g/day) of saturated fat (but not unsaturated fat) compared to individuals homozygous normal or heterozygous for this gene variant [98]. A third study indicated an interaction between this gene variant and consumption (≥ 22 g/day) of saturated fat (but not unsaturated fat) resulting in a 6.8% greater BMI compared to individuals homozygous normal or heterozygous from his gene variant in the same population [99]. And finally, the physiological basis for the APOE gene variant and saturated fat was shown to result from behavioral changes that prevented weight loss (do not skip meals) and less likelihood to exhibit protective behavior (do not plan meals) based on the modulation of plasma ghrelin [100]. A more recent study has also reported that physical activity can diminish the genetic effect of a different fat mass and obesity-associated (FTO) gene variant (rs1421085) for adiposity by 36–75% in a longitudinal multi-ethnic group consisting of 17,423 individuals [22]. Finally, the GWAS that identified the FTO gene also identified the Niemann-Pick C1 (NPC1) gene to be associated with morbid-adult obesity in European populations [7]. When this study was published, it was unknown whether the *NPC1* gene risk variants (644A > G encoding His215Arg and 2572A > G encoding Ile858Val) increased or decreased NPC1 protein function. To address this question and further investigate the NPC1 gene in relation to obesity, the BALB/cJ *Npc1* mouse model was used possessing a retroposon insertion that prematurely terminated protein translation, thereby producing a non-functional truncated NPC1 protein [101–103]. The results indicated that compared to the *Npc1* homozygous normal (Npc1^+/+^) mice, the Npc1 heterozygous (Npc1^+/-^) mice with decreased gene dosage were susceptible to weight gain when fed a high-fat diet, but not when fed a low-fat diet [104]. This study was extended using BALB/cJ-C57BL/6J hybrid Npc1^+/-^ mice that were also susceptible to weight gain and impaired glucose tolerance when fed a high-fat diet compared to hybrid Npc1^+/+^ mice fed the same diet [105]. Moreover, a subsequent study found that the C57BL/6J Npc1^+/-^ mice are susceptible to weight gain when fed a high-fat diet compared to C57BL/6J *Npc1* ^*+/+*^ mice fed the same diet [106]. An independent study has since been reported that rare human NPC1 gene loss-of-function mutations among male heterozygotes (but not female heterozygotes) have a significantly higher BMI compared to matched controls and that Npc1^+/-^ mice fed a HFD have significantly increased fat storage compared to Npc1^+/+^ mice fed the same diet [107]. The physiological basis for the Npc1 gene-nutrient interaction has recently been characterized by increased liver glycolysis and lipogenesis with an accumulation of hepatic triacylglycerol and cholesterol, in combination with decreased white adipose tissue activation of hormone-sensitive lipase and decreased triacylglycerol lipolysis [108]. In support of these results, cellular energy metabolism studies indicated that *Npc1* ^*+/-*^ fibroblasts had significantly increased glycolysis and lipogenesis in addition to significantly decreased substrate (glucose and endogenous fatty acid) oxidative metabolism resulting in an accumulation of triacylglycerol and cholesterol (Fig. 2). Finally, instead of examining just one obesity susceptibility gene and interaction with a dietary nutrient, a relatively large number of validated obesity susceptibility genes (32) were examined using a genetic risk score that was found to interact with a dietary nutrient. The first study used a combined cohort of 6934 women from the Nurses’ Health Study (NHS) and 4423 men from the Health Professionals Follow-Up Study (HPFS) which indicated an increase in relative risk of 1.19 for less than one serving per month of a sugar-sweetened beverage, 1.67 for one to four servings per month, 1.58 for two to six servings per month, and 5.06 for one or more servings per day [109]. A subsequent study using a genetic risk score and combined cohort indicated an increase in relative risk of 1.61 for less than one serving of fried food per week, 2.12 for one to three servings per week, and 2.72 for four or more servings per week [110]. In yet a third study, a genetic risk score of 32 obesity susceptibility genes was used to determine interaction with self-reported intake of various foods (healthy and unhealthy) using 18 combined cohorts of European ancestry (68,317 individuals) [21]. Interestingly, although there was no significant gene-diet interaction detected using the genetic risk score for obesity traits (BMI), the results did reveal that two genes (LRRN6C and MTIF3) for obesity traits were actually stronger for individuals consuming healthy foods.

## Nutritional and pharmacological intervention

The current literature clearly indicates that public health interventions are unable to achieve success in long-term weight loss. A basic science approach must be incorporated to more directly address behavioral and physiological reasons for the continuing obesity epidemic [111–113]. The goal for successful nutritional and pharmacological intervention of obesity depends on delineating the physiological basis for how obesity susceptibility genes promote positive energy balance and weight gain responsible for obesity. For instance, the FTO and melanocortin 4 receptor (MC4R) gene variants tend to increase preference for calorie-dense foods enriched with fat and decrease satiety. Although nutritional therapies have not yet been identified that stimulate regions of the brain (arcuate nucleus of the hypothalamus) to promote satiety for individuals harboring these gene variants, a recent study has reported that a new pharmacological agonist (setmelanotide) has been successful in producing sustained reduction in hunger and body weight (51.0 kg after 42 weeks in one patient and 20.5 kg after 12 weeks in a second patient) for patients with proopiomelanocortin deficiency as a result of treatment [114]. Moreover, other pharmacological therapies that are being used to treat obesity include orlistat (Xenical), lorcaserin hydrochloride (Belviq), phentermine and topiramate (Qsymia), bupropion and naltrexone (Contrave), and liraglutide (Saxenda). With respect to the NPC1 gene that interacts with a high-fat diet to cause dysregulation of differential energy metabolism pathways, different therapies will be required to limit hepatic lipogenesis and adipose lipolysis. Of course, for any of the obesity susceptibility genes and encoded proteins that are now being investigated, the overall goal of achieving complete efficacy for nutritional and pharmacological interventions will be modeled after the success of recombinant leptin for those individuals with congenital leptin deficiency [115]. Finally, in closing this section, it should be noted that all macronutrients (carbohydrates, protein, and fat) have unique properties that will impact regulation of energy metabolism genes and/or encoded protein to affect whole-body energy balance.

## Conclusions

The current epidemic of obesity represents a complex metabolic disease characterized in part by the interaction of obesity susceptibility gene variants with dietary nutrients. The continued investigation of gene-nutrient interactions responsible for this health problem will be important for several reasons. First, diseases such as obesity and associated complications result from undefined and complex interactions between susceptibility gene variants and various environmental factors [116]. The obesity susceptibility genes described in this review article interact with nutrients to either increase consumption of saturated fat or refined carbohydrates and to alter regulation of central metabolism pathways to increase weight [117, 118]. Second, the identification of gene-nutrient interactions should be at the forefront in attempts to understand the etiology and pathophysiology of nutrition-related diseases, particularly obesity [119]. Third, the interaction of obesity susceptibility genes with nutrients will allow for more effective individual, family, and community preventative lifestyle intervention and eventually development of targeted nutritional or medicinal therapies [120, 121]. Therefore, the overarching goal for investigating gene-nutrient interactions is to provide a plausible physiological approach to personalized nutritional or medicinal therapy that will more effectively address the current epidemic of common obesity.

## Abbreviations

APOA2: Apolipoprotein A2
BMI: Body mass index
FDA: Food and Drug Administration
FTO: Fat mass and obesity associated
GWAS: Genome-wide association study
HDL: High-density lipoprotein
HPFS: Health Professionals Follow-Up Study
MC4R: Melanocortin 4 receptor
NHS: Nurse’s Health Study
NPC1: Niemann-Pick C1
